# GADD45B Is a Potential Diagnostic and Therapeutic Target Gene in Chemotherapy-Resistant Prostate Cancer

**DOI:** 10.3389/fcell.2021.716501

**Published:** 2021-08-19

**Authors:** Qiong Wang, Wanhua Wu, Ze Gao, Kaiwen Li, Shirong Peng, Huiyang Fan, Zhongqiu Xie, Zhenghui Guo, Hai Huang

**Affiliations:** ^1^Department of Urology, Sun Yat-sen Memorial Hospital, Sun Yat-sen University, Guangzhou, China; ^2^Department of Pathology, School of Medicine, University of Virginia, Charlottesville, VA, United States; ^3^Guangdong Provincial Key Laboratory of Malignant Tumor Epigenetics and Gene Regulation, Sun Yat-sen Memorial Hospital, Sun Yat-sen University, Guangzhou, China; ^4^Department of Urology, The Sixth Affiliated Hospital of Guangzhou Medical University, Qingyuan People’s Hospital, Qingyuan, China

**Keywords:** GADD45B, chemotherapy resistance, metastatic prostate cancer, therapeutic target, MAPK pathway

## Abstract

**Background:**

Chemoresistance is the major cause of death in advanced prostate cancer (PCa), especially in metastatic PCa (mPCa). However, the molecular mechanisms underlying the chemoresistance of PCa remain unclear. Understanding the reason behind the drug resistance would be helpful in developing new treatment approaches.

**Methods:**

The Cancer Genome Atlas, Gene Expression Omnibus datasets, and clinical samples were used to examine the correlation between growth arrest and DNA damage-inducible 45 beta (GADD45B) with clinical characteristics and prognosis. Lentiviral transfection was used to construct GADD45B overexpression cell lines. Hypoxic incubator, low serum medium, or docetaxel was used to build environmental stress model or chemotherapy cell model. The MTS assay and colony formation assay were used to test cell viability. Apoptosis and cell cycle were detected by flow cytometry. The RNA and protein levels of related biomarkers were tested by Western blotting and quantitative polymerase chain reaction. Bioinformatics analysis after RNA sequencing was performed to identify the possible mechanism of how GADD45B regulates chemotherapy resistance.

**Results:**

GADD45B was related to distant metastasis but not to Gleason score, prostate-specific antigen level, T stage, or lymph node metastasis and indicated a good prognosis. The level of GADD45B increased significantly in PCa cells that faced environmental stress. It was found that a high level of GADD45B significantly enhanced the chemosensitivity. Furthermore, high GADD45B promoted cell apoptosis *via* mitogen-activated protein kinase (MAPK) pathway.

**Conclusion:**

GADD45B promoted chemosensitivity of prostate cancer through MAPK pathway. GADD45B could serve as a diagnostic biomarker and therapeutic target for mPCa or chemotherapy-resistant patients.

## Introduction

Prostate cancer (PCa) is one of the most common cancers diagnosed in men in the United States, with 191,930 new cases (accounting for 21% of all cases in men) (Siegel et al., 2020). The first-line treatment for PCa is androgen deprivation therapy, but almost all of the patients will progress to castration-resistant PCa (CRPC) within 18 to 24 months (Dong et al., 2019). Unfortunately, 84% of newly diagnosed CRPC cases have metastases (mCRPC) (Crawford et al., 2017), and the median overall survival (OS) time of mCRPC is approximately 15 months (Wülfing et al., 2019). Therapeutic resistance is the major challenge in the treatment of patients with advanced PCa, which is also one of the most important causes of mortality (Wade and Kyprianou, 2018). Therefore, there is an urgent need to elucidate the mechanisms underlying drug resistance in advanced PCa.

In the previous study, we demonstrated that Matrine inhibits the progression of PCa by promoting expression of growth arrest and DNA damage-inducible 45 beta (GADD45B) (Huang et al., 2018). GADD45B is a member of the growth arrest and DNA damage-inducible 45 (GADD45) gene family and localized at human chromosome 19p13.3. It is a structured protein with a predicted four-stranded beta-sheet core, five alpha helices, and two acidic loops (Papa et al., 2007). GADD45B has long been considered as a stress-related gene (Salvador et al., 2013; Liu et al., 2015). However, an increasing number of studies demonstrated that GADD45B has a complex function, including activation of p38 mitogen-activated protein kinase (MAPK) pathway (Xue et al., 2020), modulation of DNA demethylation (Xiao et al., 2020), and regulation of transcription (Zipperly et al., 2021). However, its role in tumor progression is controversial. GADD45B is an oncogene in ovarian cancer (Gong et al., 2021), but it is a tumor suppressor gene in liver cancer, non-small cell lung cancer, and PCa (Hori et al., 2018; Huang et al., 2018; Do et al., 2019). The mechanism by which GADD45B inhibits PCa progression is not yet fully characterized.

Here we found that GADD45B was expressed lower in metastatic tumor than localized tumor *via* databases and tissue microarray analysis. Importantly, high expression of GADD45B indicated a good prognosis. Moreover, GADD45B increased significantly when PCa cells were in a harsh environment. Further functional studies indicated that GADD45B could enhance the chemosensitivity by promoting cell apoptosis *via* the MAPK pathway. In short, we demonstrated a possible diagnostic or therapeutic target for metastatic PCa (mPCa) or chemotherapy-resistant patients.

## Materials and Methods

### Database Analysis and Clinical Tissue Samples

The Cancer Genome Atlas (TCGA) prostate adenocarcinoma datasets and two metastasis datasets from the Gene Expression Omnibus (GEO)^1^ were used to evaluate the correlation between GADD45B RNA level and clinical features of PCa. The Cancer Cell Line Encyclopedia^2^ was used to detect mRNA expression and copy number of GADD45B in different PCa cell lines. Moreover, fresh tissues of 18 PCa patients from Sun Yat-sen Memorial Hospital and 106 paraffin-embedded PCa tissues from Sun Yat-sen University Cancer Center were obtained to explore the correlation between GADD45B and clinical characteristics in RNA and protein level, respectively. We further examined the Gleason score, TNM stage, PSA (prostate-specific antigen) levels, T stage, lymph node metastasis, and distant metastasis in all clinical samples above. The use of tissues and clinical information in this study was approved by the Sun Yat-sen University’s Committees for Ethical Review of Research Involving Human Subjects (approval no. SYSEC-KY-KS-2020-201). All patients submitted their written informed consents.

### Immunohistochemistry Staining and Scoring Analyses

GADD45B (1:100; YN1622; Immunoway; Beijing, China) antibody was used to assess the protein level in the PCa samples from Sun Yat-sen University Cancer Center *via* immunohistochemistry (IHC). IHC was performed according to standard procedures as described in our previously study (Wang et al., 2017). The immunoreactivity score (IRS) was calculated according to the following formula: IRS = intensity score × percentage score; intensity score: negative = 0, weak = 1, moderate = 2, and strong = 3; percentage score: <25% = 1, 25–50% = 2, 50–75% = 3, and >75% = 4. The samples were classified as low (IRS ≤ 6) or high (IRS > 6) GADD45B expression. The IRS score was blindly quantified by two pathologists. The photographs were taken using a Nikon Eclipse 80i system (Nikon, Tokyo, Japan).

### Plasmid Construction and Stable-Transfected Cell Line

The GADD45B sequence was cloned into the pLV-CMV-EF-1a-CopGFP-T2A-puro vector (Huiyuanyuan Biotechnology, Guangzhou, China) to construct the overexpression plasmid. Package of lentivirus, infection of PCa cells, and selection of stable cells were performed as described in our previous study (Li et al., 2020b; Xie et al., 2020).

### Cell Culture and Microenvironment

Human PCa cell lines (22RV1 and DU145) and kidney cell line (293T) were purchased from ATCC. 22RV1 and DU145 cells were cultured in 1640 and Dulbecco modified Eagle medium (Gibco, United States), respectively, supplemented with 10% fetal bovine serum (FBS) (Biological Industries, United States) and 1% penicillin and streptomycin (Gibco, United States). Cells were cultured at 37°C in a humidified incubator (Thermo, Germany). For low serum stimulation, cells were cultured under 1% FBS for 24, 48, and 72 h. For hypoxia stimulation, cells were cultured under 1% O_2_ and 5% CO_2_ in a 37°C humidified incubator (Smartor 118pro, China innovation instrument, Ningbo, China) for 24 and 48 h. For docetaxel stimulation, cells were cultured with 1 nM docetaxel for 6, 12, and 48 h.

### RNA Isolation and Quantitative Polymerase Chain Reaction

Total RNA from cells was isolated using Trizol reagent (TaKaRa Biotechnology, Dalian, China) as previously described (Xie et al., 2019; Li et al., 2020a). Total RNA from clinical samples was isolated according to standard procedures. In brief, frozen tissue samples were weighed and processed on dry ice to prevent thawing. Five to 10 mg of tissue was added to the mortar liquid nitrogen precooled mortar. The tissues were grinded for 10 min, and 5 to 8 mL of liquid nitrogen was added every 1 min to keep the mortar cool. Then total RNA was isolated from the grated tissues by Trizol according to standard procedure.

### Protein Extracted and Western Blotting

The proteins from cell samples were harvested using RIPA (Radio-Immune Precipitation Assay) lysis buffer (Beyotime, Nanjing, China) and quantified by bicinchoninic acid protein assay kit (Beyotime Biotechnology, Shanghai, China). Western blotting (WB) was performed by 10% sodium dodecyl sulfate polyacrylamide gel electrophoresis as previously described (Xiao et al., 2017). Primary antibodies specific to GADD45B (1:500; YN1622; Immunoway; Beijing, China), P38 (1:1,000; 8690S; CST, Danvers, MA, United States), p-p38 (1:1,000; 4511S; CST, Danvers, MA, United States), and GAPDH (1:1,000; 97166S; CST, Danvers, MA, United States) were used. The membranes were then incubated with antirabbit (cw0103s, 1:5,000; Cwbiotech, Beijing, China) or anti-mouse (cw0102s, 1:5,000; Cwbiotech) secondary antibody for 1 h at room temperature. We detected protein band signals by using Immobilon Western Chemiluminescent HRP Substrate (WBKLS0500, Darmstadt, Germany).

### Cytotoxicity Assay, Colony Formation Assay, and Migration Assay

For cytotoxicity assay, the MTS assay (Promega, Beijing, China) was used to test the viability of 22RV1 and DU145 cells treated with docetaxel (Selleck, Shanghai, China). In brief, cells (1,000 for DU145 and 2,000 for 22RV1) were seeded in 96-well plates with different concentrations of docetaxel and cultured for 72 h. Then, we calculated the IC50 according to the absorbance at 492 nm. For colony formation assay, 1,500 DU145 cells were seeded in six-well plates and cultured in incubator for 10 days to form macroscopic clones. After staining with 0.1% crystal violet, we counted the number of colonies in different groups.

The 24-well Transwell chamber (8 μM, 353097; Corning, Glendale, AZ, United States) was used for the migration assay. In brief, 40,000 cells in 200 μL of 1% FBS medium were seeded in the top insert chamber, and 600 μL of medium containing 10% FBS was added into the lower chamber. The top chamber was fixed with 4% paraformaldehyde and stained with 0.2% crystal violet after 12 h incubation. The migrated cells on the lower membrane surface of the top chamber were detected under a microscope (Nikon, Tokyo, Japan).

### Flow Cytometry

For apoptosis test, 5 × 10^5^ cells were washed twice with chilled phosphate-buffered saline (PBS) before being resuspended in 300 μL 1 × binding buffer. Then, 5 μL annexin V–APC and 10 μL 7-AAD (abs50008; Absin, Shanghai, China) were added into each testing tube. Samples were incubated in the darkroom for 5 min at room temperature following by detection with Flow cytometry (Beckman CytoFLEX, United States). CytExpert 2.0 software was used to analyze the proportion of apoptosis.

For cell cycle test, the same number of cells were washed twice with chilled PBS and then fixed with precooled 70% ethanol at 4°C for 16 h. Cells were resuspended with 50 μL RNase and 300 μL propidium iodide and incubated in the darkroom for 20 min following by flow cytometric analysis. ModFit software was used to analyze the ratio of G1, S, and G2 phases in cells.

### RNA Sequencing and Data Analysis

GADD45B overexpression and its control group of DU145 cells were sent for RNA sequencing (Ranshi Biotechnology, Guangzhou, China). RNA samples were sent to RiboBio Co., Ltd., Guangzhou, China, for next-generation sequencing. The reagents provided in an Illumina^®^ TruSeq^®^ Stranded Total RNA library prep workflow were used to convert total RNA into a library of template molecules of known strand origin. Sequencing and clustering of cDNA libraries were carried out on the NovaSeq 6000 System. Genes with log2 (fold change) ≥1 or ≤−1 were considered significantly differentially expressed. And the RNA-seq results have been uploaded to ArrayExpress (E-MTAB-10704, RNA-seq of human PCa cell line DU145 overexpressed GADD45B against control). Gene Ontology (GO) term analysis^3^ and Gene Set Enrichment Analysis (GSEA)^4^ were performed for the significantly differentially expressed genes in our RNA-seq dataset or TCGA dataset.

### Statistical Analysis

All quantitative data were assessed by one-way analysis of variance followed by Student *t* test (GraphPad, La Jolla, CA, United States). Pearson *χ*^2^ and Fisher exact tests were used to analyze the correlation of GADD45B with clinical features. The Kaplan–Meier method was used to describe biochemical recurrence–free survival in those patients from Sun Yat-sen University Cancer Center. *p* < 0.05 was considered statistically significant after SPSS 22.0 analysis.

## Results

### GADD45B Was Lowly Expressed in mPCa and Predicted a Good Prognosis

**FIGURE 1:**
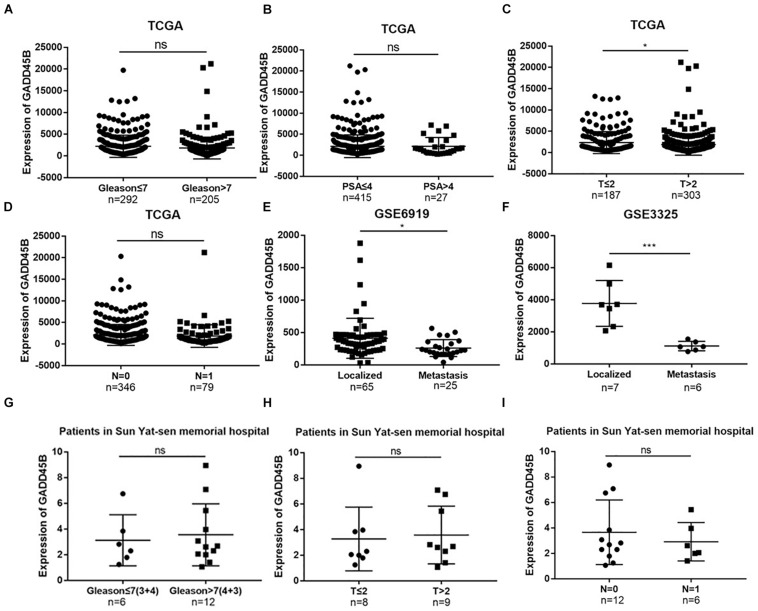
TCGA, GEO database, and clinical samples identified GADD45B correlated with the metastasis of PCa. **(A–D)** Correlation of GADD45B expression with clinical features of PCa patients in the TCGA dataset. **(E,F)** Correlation of GADD45B expression with metastatic PCa datasets from GEO. **(G–I)** Correlation of GADD45B expression with clinical features of PCa patients from Sun Yat-sen Memorial Hospital. *p < 0.05, ***p < 0.001, and not statistically significant *p* > 0.05.

In the previous study, we found that Matrine inhibits the progression of PCa by promoting expression of GADD45B (Huang et al., 2018). However, the mechanism underlying the role of GADD45B in affecting PCa remains unclear. In this study, we employed TCGA dataset and GEO datasets (GSE6919 and GSE3325) to explore the relationship between the expression of GADD45B and clinicopathologic characteristics of PCa. The results showed that a high RNA level of GADD45B was related to a low T stage (*p* = 0.042) but not to Gleason score, PSA level, and lymph node metastasis (*p* > 0.05, Figures 1A–D). In addition, low level of GADD45B indicated a high potential for distant metastasis in GSE6919 (*p* = 0.020, Figure 1E) and GSE3325 (*p* = 0.001, Figure 1F). Furthermore, the clinical samples from our hospital demonstrated that the level of GADD45B was not related to Gleason score, T stage, and lymph node metastasis (*p* > 0.05, Figures 1G–I).

**FIGURE 2:**
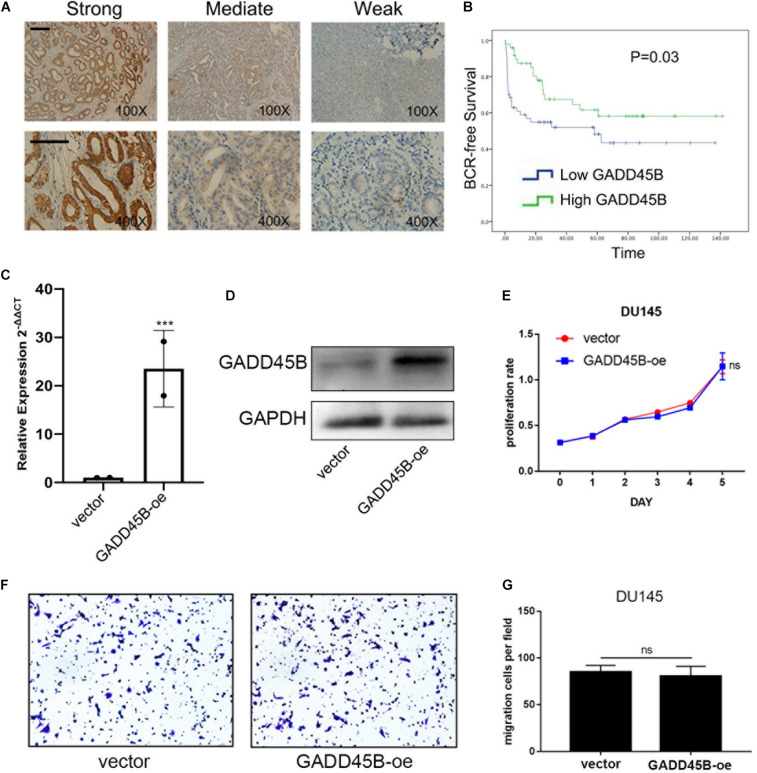
GADD45B predicted a good prognosis and had no effect on the proliferation and migration of PCa cells. **(A)** Representative immunohistochemical staining and quantification showed the expression level of GADD45B in PCa tissues of patients from Sun Yat-sen University Cancer Center. Magnification 100×, scale bar = 200 μm, Magnification 400×, scale bar = 100 μm. **(B)** Biochemical recurrence survival analysis of GADD45B in patients form Sun Yat-sen University Cancer Center. **(C,D)** RT-PCR and WB analysis of GADD45B levels in GADD45B-overexpressing DU145 cell line. **(E)** The MTS assay test of cell viability of DU145 cells with GADD45B overexpression. **(F,G)** Representative images and histogram analysis of migration assays after upregulation of GADD45B. ***p < 0.001 and not statistically significant *p* > 0.05.

It is well known that mRNA levels are not sufficient to predict protein levels in many scenarios (Liu et al., 2016). Thus, we further analyzed the protein level of GADD45B in a large-scale sample cohort containing 106 PCa specimens (Figure 2A). Statistical analyses showed that a low protein level of GADD45B implied a higher potential for distant metastasis (*p* = 0.001, Table 1). Similarly, the GADD45B protein level was not related to age, Gleason score, PSA level, T stage, or lymph node metastasis (*p* > 0.05). More importantly, Kaplan–Meier survival indicated that high protein levels of GADD45B mean a better prognosis in PCa (*p* = 0.03, Figure 2B). These results suggested that high GADD45B was associated with a smaller possibility of distant metastasis and a better prognosis.

**TABLE 1:** Correlation of GADD45B expression with clinico-pathologic characteristics of PCa patients in Sun Yat-sen University Cancer Center (106 patients).

Clinical features	Total patients, n	Low, n (%)	High, n (%)	*P-*value
**Age, years**				
≤ 65	51	25(49.0%)	26(51.0%)	0.344
>65	55	32(58.2%)	23(41.8%)	
***Gleason score***				
≤7 (3+4)	43	21(48.8%)	22(51.2%)	0.400
≥7 (4+3)	63	36(57.1%)	27(42.9%)	
**Serum PSA levels, ng/ml**				
≤20	65	34(52.3%)	31(47.7%)	0.560
>20	36	21(58.3%)	15(41.7%)	
**T stage**				
T1–T2	19	10(52.6%)	9(47.4%)	0.379
T3–T4	26	17(65.4%)	9(34.6%)	
**Lymph node metastasis**				
N0	43	19(44.2%)	24(55.8%)	0.107
N1	30	19(63.3%)	11(36.7%)	
**Distant metastasis**				
M0	26	6(23.1%)	20(76.9%)	0.001**
M1	24	17(70.8%)	7(29.2%)	

Cut off value of Immuno-reactivity score: 6 (≥6, High; <6, Low.); T, tumor; N, node; M, metastasis. **P < 0.01.

### GADD45B Had No Effect on the Proliferation and Migration of PCa Cells

DU145 is a naturally mPCa cell line that was isolated from a lesion in the brain of a patient with metastatic prostate carcinoma. We constructed a stable GADD45B overexpressed DU145 cell line by using pLV-CMV-EF-1a-CopGFP-T2A-puro vector to investigate if GADD45B could affect the proliferation and migration of PCa cells. Reverse transcriptase–polymerase chain reaction (RT-PCR) and WB showed that the mRNA and protein levels of GADD45B were increased (Figures 2C,D). Further MTS assay and Transwell assay showed that overexpressed GADD45B did not influence the proliferation and migration of DU145 (Figures 2E–G). These results suggested that GADD45B improved the prognosis of PCa, not by directly affecting its proliferation or metastasis.

### GADD45B Increased Significantly When Facing Environmental Stresses

**FIGURE 3:**
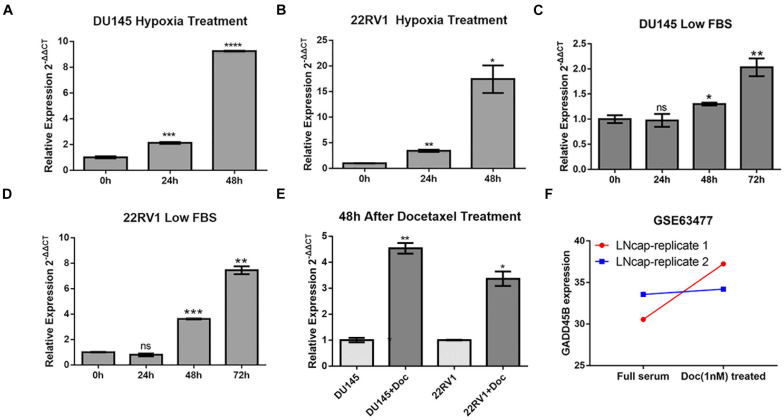
GADD45B increased significantly in cells with environmental stresses and docetaxel treatment. RT-PCR analysis of GADD45B levels in DU145 and 22RV1 cells with hypoxia **(A,B)**, low serum **(C,D)**, and docetaxel stimulation **(E)**. **(F)** The expression of GADD45B in a docetaxel treatment dataset. *p < 0.05, **p < 0.01, ***p < 0.001, ****p < 0.0001, and not statistically significant *p* > 0.05.

Compared with localized tumor, metastatic tumor behaves two obvious characteristics: one is adhesion and motility-related pathways activation (such as Wnt, transforming growth factor pathways), which leads to distant organ metastasis through lymph or blood (Robert, 2013). The other is increased environmental tolerance, which ensures tumor proliferation when facing a stressed microenvironment or even clinical treatment (Norouzi et al., 2018; Hou et al., 2020). Given that GADD45B is an environmental stress–related gene and it does not directly affect PCa metastasis, we suspected that this gene may be involved in the environmental tolerance and treatment resistance of mPCa. Therefore, we further chose 22RV1 and DU145 to explore if GADD45B plays a role when facing harsh microenvironment. The results showed that GADD45B was significantly increased when PCa cells were faced with hypoxia, low serum, or docetaxel (Figures 3A–E). Similarly, another study also found GADD45B increased when facing docetaxel treatment in another PCa cell line LNcap (GSE63477, Figure 3F). These results demonstrated that alteration of GADD45B was related to environment stresses and clinical treatment, suggesting that low expression of GADD45B may promote the tolerance of mPCa to harsh microenvironments and chemotherapy, which in turn leads to tumor progression.

### GADD45B Was Correlated With Chemotherapy Sensitivity–Related Genes and Lowly Expressed in Docetaxel-Resistant PCa Cell Lines

**FIGURE 4:**
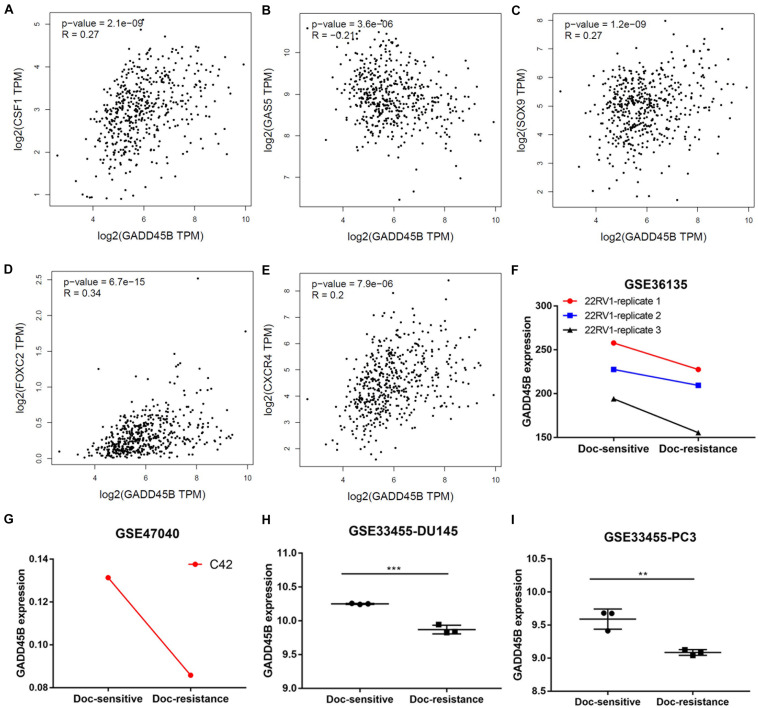
GADD45B was correlated to chemotherapy sensitivity–related genes and lowly expressed in docetaxel-resistant PCa cell lines. **(A–E)** The correlation between GADD45B and the reported chemotherapy sensitivity–related genes: CSF1 **(A)**, GAS5 **(B)**, SOX9 **(C)**, FOXC2 **(D)**, and CXCR4 **(E)**. **(F–I)** GADD45B was lowly expressed in docetaxel-resistant 22RV1 **(F)**, C42 **(G)**, DU145 **(H)**, and PC3 **(I)**. **p < 0.01 and ***p < 0.001.

Chemotherapy resistance is one of the important characteristics of mPCa, and it is also an important cause of patient death (Wade and Kyprianou, 2018). To explore if GADD45B was related to chemotherapy sensitivity of PCa, we first used GEPIA to depict the correlation between GADD45B and chemotherapy sensitivity–related genes. We found that GADD45B was significantly related to genes of CSF1 (Guan et al., 2021), GAS5 (Ashrafizaveh et al., 2021), SOX9 (Song et al., 2016), FOXC2 (Gong et al., 2020), and CXCR4 (Wang et al., 2021) (*p* < 0.05, Figures 4A–E). More importantly, the GEO datasets analysis also showed that GADD45B was lowly expressed in those docetaxel resistance 22RV1, C42, DU145, and PC3 cell lines (Figures 4F–I). The results suggested that GADD45B is likely to be involved in the chemotherapy resistance process of PCa.

### Overexpressed GADD45B Facilitated Chemotherapy Sensitivity of PCa Cells

**FIGURE 5:**
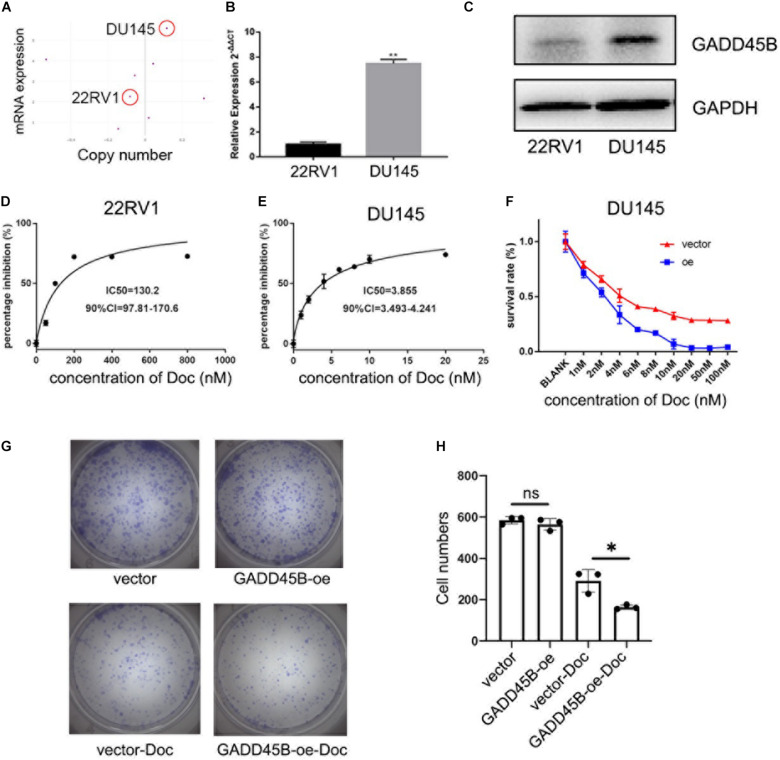
GADD45B facilitated chemotherapy sensitivity of PCa cells. **(A)** mRNA expression and gene copy number of GADD45B in prostate-related cell lines from CCLE. **(B,C)** RT-PCR and WB analysis of GADD45B levels in DU145 and 22RV1 cell lines. **(D,E)** The MTS assay on the IC50 to docetaxel in DU145 and 22RV1 cells. **(F)** The MTS assay on the cell survival upon docetaxel treatment in GADD45B overexpressed cells. **(G,H)** Representative images and histogram analysis of colony formation assay before and after docetaxel treatment. *p < 0.05, **p < 0.01, and not statistically significant *p* > 0.05.

To explore if overexpression of GADD45B could enhance the chemotherapy sensitivity of PCa, we used CCLE to compare the level of GADD45B in different PCa cell lines. Results showed that DU145 possessed both higher mRNA expression and gene copy number of GADD45B than 22RV1 (Figure 5A). Further, RT-PCR and WB also validated that DU145 had a higher GADD45B level than 22RV1 in both transcription and protein levels (Figures 5B,C). Importantly, cytotoxicity assay demonstrated that the IC50 to docetaxel in DU145 was 3.855 nM, which was much lower than that in 22RV1 (130.2 nM, Figures 5D,E). In addition, further cytotoxicity assay and colony formation assay showed that overexpressed GADD45B could alleviate the chemotherapy resistance in DU145 significantly (Figures 5F–H). These results suggested that GADD45B increased the inhibitory effect of chemotherapy on the proliferation of PCa.

### GADD45B Facilitated Chemotherapy Sensitivity by Regulating Cell Apoptosis

**FIGURE 6:**
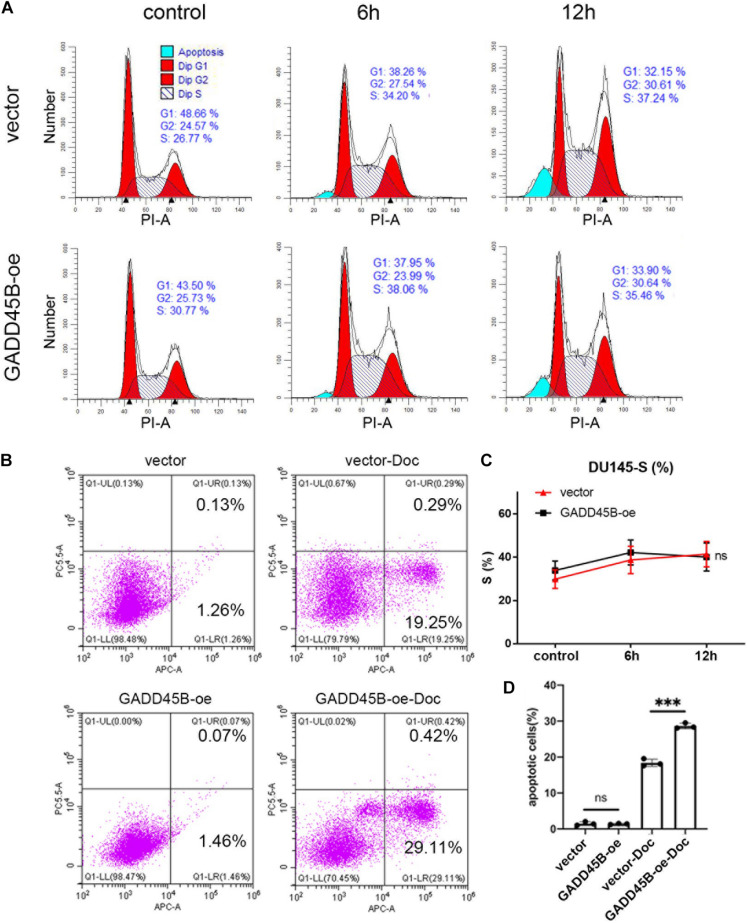
GADD45B promoted chemotherapy sensitivity by regulating cell apoptosis. **(A,C)** Representative flow cytometry cell cycle images and statistical analysis of DU145 cells before and 6 and 12 h after chemotherapy. **(B,D)** Representative images and histogram analysis of flow cytometry on apoptosis of DU145 cells before and 48 h after chemotherapy. ***p < 0.001 and not statistically significant *p* > 0.05.

Apoptosis and blockage of cell cycle are two important factors affecting cell proliferation. Therefore, flow cytometry was performed to test the change of cell cycle and apoptosis before and after docetaxel treatment. For cell cycle, PCa cells underwent S phase arrest after receiving docetaxel therapy, but no difference was found between GADD45B overexpressed and control cells (Figures 6A,C). Thus, we suspected that GADD45B may facilitate chemotherapy sensitivity by regulating cell apoptosis. Further results showed that although docetaxel caused a certain degree of apoptosis, overexpression of GADD45B induced a higher level of apoptosis (Figures 6B,D). In addition, overexpression of GADD45B alone has no effect on cell cycle or apoptosis. Thus, we believed GADD45B facilitated chemotherapy sensitivity by promoting cell apoptosis.

### GADD45B Regulates Cell Apoptosis Through p38/MAPK Pathway

**FIGURE 7:**
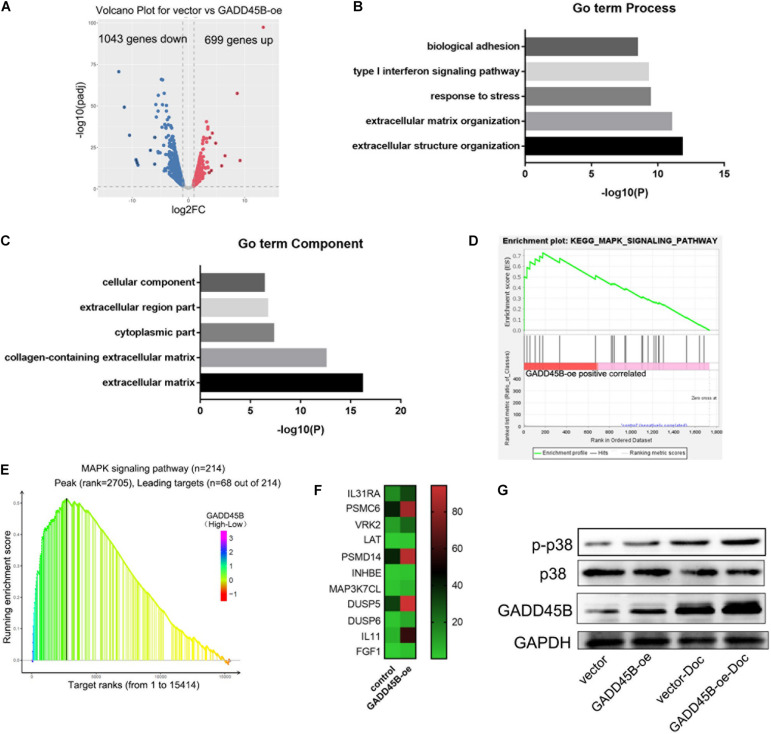
GADD45B regulated cell apoptosis through the p38/MAPK pathway. **(A)** Volcano map of differentially expressed genes in GADD45B overexpression group. **(B,C)** GO term analysis of differentially expressed genes in GADD45B overexpressed cells. **(D)** GSEA analysis of differentially expressed genes in GADD45B overexpressed cells. **(E)** GSEA analysis of GADD45B in TCGA. **(F)** Heatmap of MAPK pathway–related genes when GADD45B overexpressed. **(G)** Representative image of the WB of GADD45B, p38 and phosphorylated p38 protein levels before and 48 h after chemotherapy in DU145 cells.

To explore how GADD45B affects apoptosis in PCa, the GADD45B overexpressed DU145 cells were used for RNA sequencing. The results showed that 699 genes were upregulated, and 1,042 genes were downregulated in GADD45 overexpressed cells (fold change ≥2, Figure 7A). GO term analysis and KEGG pathway enrichment analysis were performed to further explore the roles of these genes. GO term analysis demonstrated that cellular component were important for metastasis and stress response (Figures 7B,C). Further KEGG pathway enrichment analysis indicated GADD45B was related to MAPK pathways (Figure 7D). Similarly, we performed GSEA analysis on the TCGA database and found that MAPK pathway was enriched when GADD45B was overexpressed in cells (Figure 7E). Many MAPK pathway–related genes were also up-regulated when GADD45B was overexpressed (Figure 7F). Considering that p38/MAPK is an important upstream of apoptosis (Fang et al., 2020; Wu et al., 2020), GADD45B might regulate apoptosis by activating p38/MAPK pathway, thus facilitating chemotherapy sensitivity. WB was performed, and it was found that p-p38 was upregulated after docetaxel treatment in DU145 cells. GADD45B could promote the phosphorylation of p38, and overexpressed GADD45B could facilitate this process to a greater extent when facing docetaxel treatment (Figure 7G). These results indicated that GADD45B may affect cell apoptosis through p38/MAPK signaling pathway, thus regulating chemotherapy sensitivity.

## Discussion

Metastatic advanced PCa is one of the important causes of patient death. Here, we found that GADD45B was significantly down-regulated in mPCa, and it was associated with the prognosis of PCa. Further study demonstrated that loss of GADD45B contributed to the adaptation of the harsh environment. Moreover, it affected cell apoptosis potentially by regulating the p38/MAPK pathway and ultimately promoting the sensitivity of PCa to chemotherapy.

Docetaxel is an inhibitor of depolymerization of microtubules by binding to stabilized microtubules and is the first chemotherapeutic agent to improve OS in men with advanced PCa (Barata and Sartor, 2019). Docetaxel was supposed to block cell cycle and induce apoptosis in many studies (Deng et al., 2021; Markowitsch et al., 2021). Cell cycle arrest is often used by cells to repair damage before cell proliferation. However, if the damage is too severe, the cell cycle will be arrested for a long time, and other signaling mechanisms will activate to induce cell apoptosis. Compared to control group, overexpression of GADD45B had no effect on the cell cycle blocking even facing chemotherapy, suggesting that GADD45B promoted chemotherapy sensitivity by promoting apoptosis directly.

GADD45B is reported to bind and activate MTK1/MEKK4 kinase, which is an upstream activator of p38 (Tsuchimochi et al., 2010; Liao et al., 2020). Here we also found that overexpression of GADD45B could facilitate phosphorylation of p38 under docetaxel treatment. Considering that p38/MAPK pathway is an important upstream of apoptosis (Fang et al., 2020; Wu et al., 2020), we suggest that GADD45B promotes cell apoptosis through p38/MAPK pathway. It should be noted that overexpression of GADD45B itself did not cause cell apoptosis, even though it could activate p38/MAPK. Only when facing chemotherapy can it promote cell apoptosis. Therefore, we suspect that slight activation of p38/MAPK pathway cannot cause cell apoptosis, but there may be a positive feedback regulation after overexpression of GADD45B.

Additionally, GADD45B is either an oncogene or tumor suppressor gene in different cancers (Hori et al., 2018; Huang et al., 2018; Do et al., 2019; Gong et al., 2021). Its roles in PCa are still not fully clarified. Here, we found one possible mechanism that GADD45B improves the prognosis of PCa. What interests us more is that GADD45B could influence nuclear factor κB pathway, thus enhancing antitumor immune responses (Bubici et al., 2004; Wang et al., 2006). Therefore, loss of GADD45B may be one of the reasons why PCa resists immunotherapy.

A limitation of this study is that we only constructed GADD45B overexpressed system in PCa cells. We have designed five siRNAs to knock down this gene, but we have not observed the corresponding knockdown effects (Supplementary Figure 1 and Supplementary Table 1). The off-target effect was a possible reason leading to this. Further, the Cas9 or Cas13a technique may be useful to establish a knockdown system. In addition, we believe that the role of GADD45B in promoting the prognosis should not be limited to promoting chemotherapy sensitivity, because many studies have shown other effects, such as tumor immunity. Future studies should focus on evaluating the role of GADD45B in immunotherapy and other therapies.

## Conclusion

GADD45B promoted chemosensitivity of PCa potentially *via* MAPK signaling pathway. GADD45B could serve as a diagnostic or therapeutic target for mPCa or chemotherapy-resistant patients.

## Data Availability Statement

The RNA-seq data presented in the study are deposited in the ArrayExpress repository (https://www.ebi.ac.uk/arrayexpress/), accession number (E-MTAB-10704).

## Ethics Statement

The studies involving human participants were reviewed and approved by Sun Yat-sen University’s Committees for Ethical Review of Research Involving Human Subjects. The patients/participants provided their written informed consent to participate in this study.

## Author Contributions

HH, ZG, and ZX designed the study and guided the whole experiment. QW and WW were the main participants in the experiment. HH and QW wrote the manuscript and performed the data analysis. ZG and KL participated in bioinformatics analysis. SP and HF performed the immunohistochemistry (IHC) experiments. ZG and ZX critically revise the draft for important intellectual content.

## Conflict of Interest

The authors declare that the research was conducted in the absence of any commercial or financial relationships that could be construed as a potential conflict of interest.

## Publisher’s Note

All claims expressed in this article are solely those of the authors and do not necessarily represent those of their affiliated organizations, or those of the publisher, the editors and the reviewers. Any product that may be evaluated in this article, or claim that may be made by its manufacturer, is not guaranteed or endorsed by the publisher.

## Footnotes

1
https://www.ncbi.nlm.nih.gov/geo/query/acc.cgi

2
https://portals.broadinstitute.org/ccle

3
http://cbl-gorilla.cs.technion.ac.il/

4
http://www.gsea-msigdb.org

## Supplementary Material

Supplementary Figure 1 RT-PCR analysis of GADD45B levels in DU145 with five different siRNAs.
