# Supplementary material for: GADD45B Is a Potential Diagnostic and Therapeutic Target Gene in Chemotherapy-Resistant Prostate Cancer
Source: Front Cell Dev Biol. 2021 Aug 19;9:716501. doi: 10.3389/fcell.2021.716501 (PMC8417000; doi:10.3389/fcell.2021.716501)
Supplement: Supplementary file 1 [file Table_1.docx]

**Table S1The sequences of primersand siRNAs used in this study.**

**Primers**

| **Name** | | **Forward (5'-3')** | | **Reverse(5'-3')** | |
| --- | --- | --- | --- | --- | --- |
| **GADD45B** | **CCTGCAAATCCACTTCACGC** | | **GTGTGAGGGTTCGTGACCAG** | |  |
| **GAPDH** | **CAAGGCTGAGAACGGGAAG** | | **TGAAGACGCCAGTGGACTC** | |  |

**siRNAs**

| **Name** | | **sense（5'-3'）** | | **antisense（5'-3'）** | |
| --- | --- | --- | --- | --- | --- |
| siGADD45B-1 | CCGUUGGUUUCCGCAACUUTT | | AAGUUGCGGAAACCAACGGTT | |  |
| siGADD45B-2 | GCCAAGGACUUUGCAAUAUTT | | AUAUUGCAAAGUCCUUGGCTT | |  |
| siGADD45B-3 | GCCGGUGUUUGUCAACAAATT | | UUUGUUGACAAACACCGGCTT | |  |
| siGADD45B-4 | GCCAGCUACUGCGAAGAAATT | | UUUCUUCGCAGUAGCUGGCTT | |  |
| siGADD45B-5 | CCUCGACAAGACCACACUUTT | | AAGUGUGGUCUUGUCGAGGTT | |  |
