# Supplementary figures and images for: GADD45B Is a Potential Diagnostic and Therapeutic Target Gene in Chemotherapy-Resistant Prostate Cancer
Source: Front Cell Dev Biol. 2021 Aug 19;9:716501. doi: 10.3389/fcell.2021.716501 (PMC8417000; doi:10.3389/fcell.2021.716501)

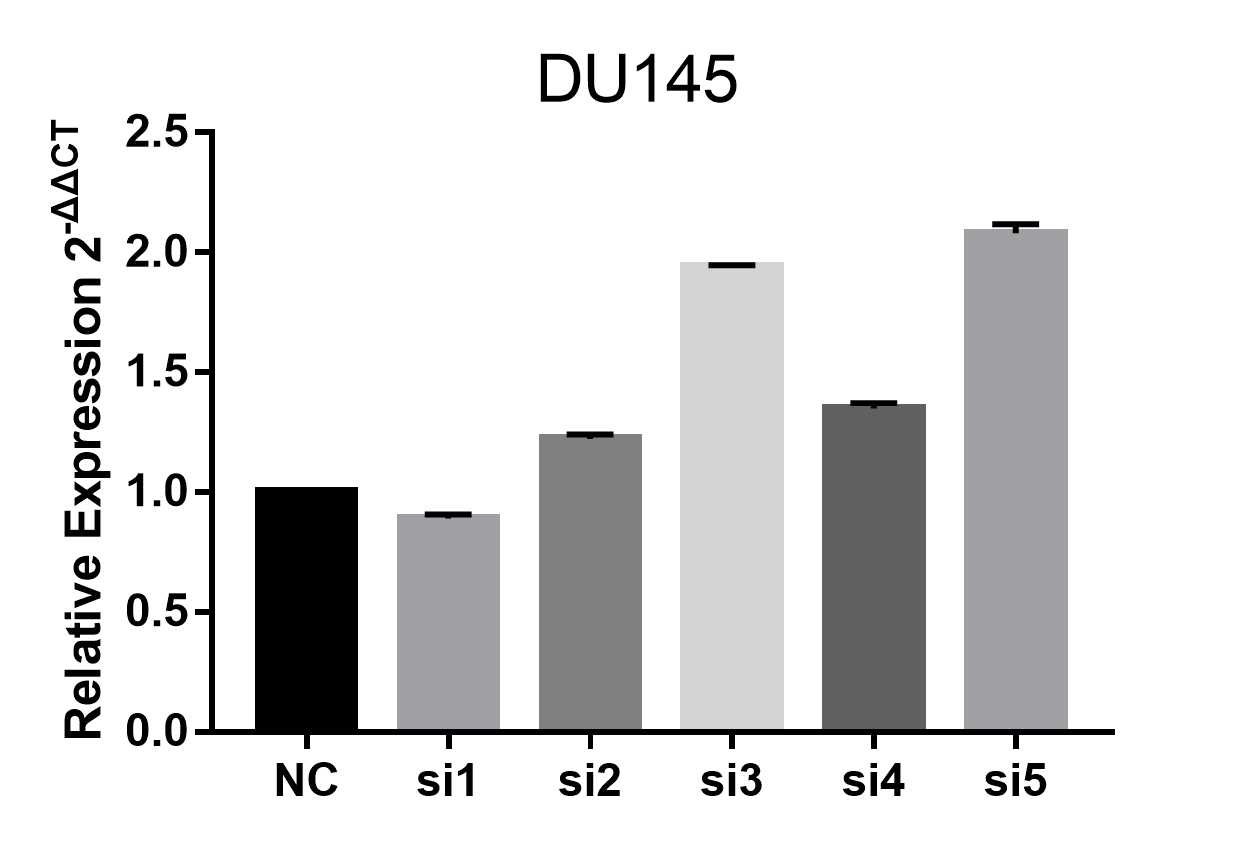

Supplement: Supplementary Figure 1 — RT-PCR analysis of GADD45B levels in DU145 with five different siRNAs. [file Image_1.TIF]
